# An Updated Review of the Diagnostic Methods in Delayed Drug Hypersensitivity

**DOI:** 10.3389/fphar.2020.573573

**Published:** 2021-01-12

**Authors:** Ana Copaescu, Andrew Gibson, Yueran Li, Jason A. Trubiano, Elizabeth J. Phillips

**Affiliations:** ^1^ Department of Infectious Diseases, Austin Health, Center for Antibiotic Allergy and Research, Heidelberg, VIC, Australia; ^2^ Immunology and Infectious Diseases, Murdoch University, Murdoch, WA, Australia; ^3^ Clinical Immunology and Allergy, McGill University Health Center, Montréal, Canada; ^4^ Department of Oncology, Sir Peter MacCallum Cancer Center, The University of Melbourne, Parkville, VIC, Australia; ^5^ Department of Medicine (Austin Health), The University of Melbourne, Heidelberg, VIC, Australia; ^6^ The National Center for Infections in Cancer, Peter MacCallum Cancer Center, Melbourne, VIC, Australia; ^7^ Institute for Immunology and Infectious Diseases, Murdoch University, Murdoch, WA, Australia; ^8^ Department of Infectious Diseases, Vanderbilt University Medical Center, Nashville, TN, United States

**Keywords:** delayed hypersensitivity reaction, drug allergy, severe cutaneous adverse reactions, T cells, skin testing, lymphocyte transformation test (LTT), enzyme linked ImmunoSpot (ELISpot), HLA

## Abstract

Delayed drug hypersensitivity reactions are clinically diverse reactions that vary from isolated benign skin conditions that remit quickly with no or symptomatic treatment, drug discontinuation or even continued drug treatment, to the other extreme of severe cutaneous adverse reactions (SCARs) that are associated with presumed life-long memory T-cell responses, significant acute and long-term morbidity and mortality. Diagnostic “in clinic” approaches to delayed hypersensitivity reactions have included patch testing (PT), delayed intradermal testing (IDT) and drug challenges for milder reactions. Patch and IDT are, in general, performed no sooner than 4–6 weeks after resolution of the acute reaction at the maximum non-irritating concentrations. Functional *in vitro* and *ex vivo* assays have largely remained the province of research laboratories and include lymphocyte transformation test (LTT) and cytokine release enzyme linked ImmunoSpot (ELISpot) assay, an emerging diagnostic tool which uses cytokine release, typically IFN-γ, after the patient’s peripheral blood mononuclear cells are stimulated with the suspected drug(s). Genetic markers such as human leukocyte antigen have shown recent promise for both pre-prescription screening as well as pre-emptive and diagnostic testing strategies.

## Introduction

In this review, we will address the immune mechanisms of delayed hypersensitivity and how they have formed the premise for diagnostic methods used in the clinic and research laboratory such as intradermal skin testing (IDT), patch testing (PT) and new and investigational laboratory-based methods such as the lymphocyte transformation test (LTT) and the enzyme linked ImmunoSpot (ELISpot) assay. In addition, the role of genetic markers such as human leukocyte antigen (HLA) in screening, early diagnosis and diagnosis will be discussed.

## Delayed Hypersensitivy Reactions

Delayed drug hypersensitivities are predominantly the result of T-cell mediated reactions of varying severity and clinical diagnosis such as maculopapular exanthema (MPE), fixed drug eruption (FDE), symmetrical drug-related intertriginous and flexural exanthema (SDRIFE), single organ disease (e.g., drug induced liver injury (DILI) and kidney diseases), acute generalized exanthematous pustulosis (AGEP), drug reaction with eosinophilia and systemic symptoms (DRESS), Stevens-Johnson syndrome (SJS) and toxic epidermal necrolysis (TEN).

MPE or morbilliform drug eruption is the most common of the self-limiting reactions to drugs characterized by erythematous macules and papules that can become generalized and confluent and are associated with pruritis and/or mild eosinophilia (Peter et al., 2017). FDE is characterized by red dark lesions localized in the same area after drug re-exposure that might be accompanied by a burning or itchy sensation (Rive et al., 2013). SDRIFE is characterized by a well-demarcated macular eruption involving the flexural or intertriginous folds, inguinal and peri-genital as well as gluteal and peri-anal areas (Wolf and Tuzun, 2015). DILI generally manifests as an isolated hepatitis with multiple metabolic, immune and genetic factors considered causal (Rive et al., 2013). However, some patients can present with features of hypersensitivity such as fever and skin eruption as well as pruritus with secondary excoriations. AGEP is a non-follicular sterile pustular eruption over widespread erythema, with a predilection for the flexural folds, and accompanied by fever and/or biological abnormalities (Peter et al., 2017). A validation score from the EuroSCAR group criteria can be used to confirm the clinical diagnostic for AGEP cases (Sidoroff et al., 2001). The main clinical features of DRESS or drug-induced hypersensitivity syndrome (DIHS) are erythematous urticaria-like plaques or violaceous skin eruption that can progress to exfoliative dermatitis, facial and extremity edema, lymphadenopathy, fever, biological abnormalities and internal organ involvement. The European Registry of Severe Cutaneous Adverse Reactions (RegiSCAR) score is calculated using clinical and laboratory data to determine the likelihood of disease (definite, probable, possible or no case) (Kardaun et al., 2007). Another multisystem disease related to drug exposure is the abacavir hypersensitivity syndrome (AB HS) that is characterized by constitutional symptoms including fever, gastrointestinal manifestations and skin eruption (Clay, 2002; Phillips et al., 2002).

SJS and TEN are characterized by skin detachment and full-thickness epidermal necrosis of various severities depending of the body surface area (BSA) affected (1–10% for SJS, 10–30% for SJS/TEN overlap and >30% for TEN) as well as blistering of mucous membranes accompanied by other serious systemic manifestations (Peter et al., 2017). As the mortality can reach 30–50% (Rive et al., 2013), a validated clinical score of toxic epidermal necrosis (SCORTEN), can be calculated at admission to predict mortality (Bastuji-Garin et al., 2000). Drug causality can be assessed with the algorithm of drug causality for epidermal necrolysis (ALDEN) score, an algorithm that helps identify the most likely causal drug(s) based on criteria such as type of drug, timing and possible alternative causes (Shear and Dodiuk-Gad, 2019). The time from the drug exposure to the development of symptoms can vary from 4 to 28 days and, in one third of cases, no causal agent is identified (Duong et al., 2017). The spectrum of T-cell mediated phenotypic scoring tools are outlined in Table 1.

**TABLE 1:** Clinical diagnosis and described scoring algorithms.

Clinical diagnosis	AGEP	DRESS	SJS/TEN
Disease likelihood	AGEP validation score	RegiSCAR score	None
Drug causality	Naranjo score	Naranjo score	ALDEN score
			Naranjo score
Mortality	None	None	SCORTEN

AGEP, acute generalized exanthematous pustulosis; DRESS, drug reaction with eosinophilia and systemic symptoms; SJS/TEN, SJS/TEN, Stevens-Johnson syndrome/toxic epidermal necrolysis. ALDEN, algorithm of drug causality for epidermal necrolysis; Naranjo score: The Adverse Drug Reaction (ADR) Probability Scale; RegiSCAR, european registry of severe cutaneous adverse reactions; SCORTEN, score of toxic epidermal necrosis.

### Mechanisms of Immune Response in Delayed Hypersensitivities

**FIGURE 1:**
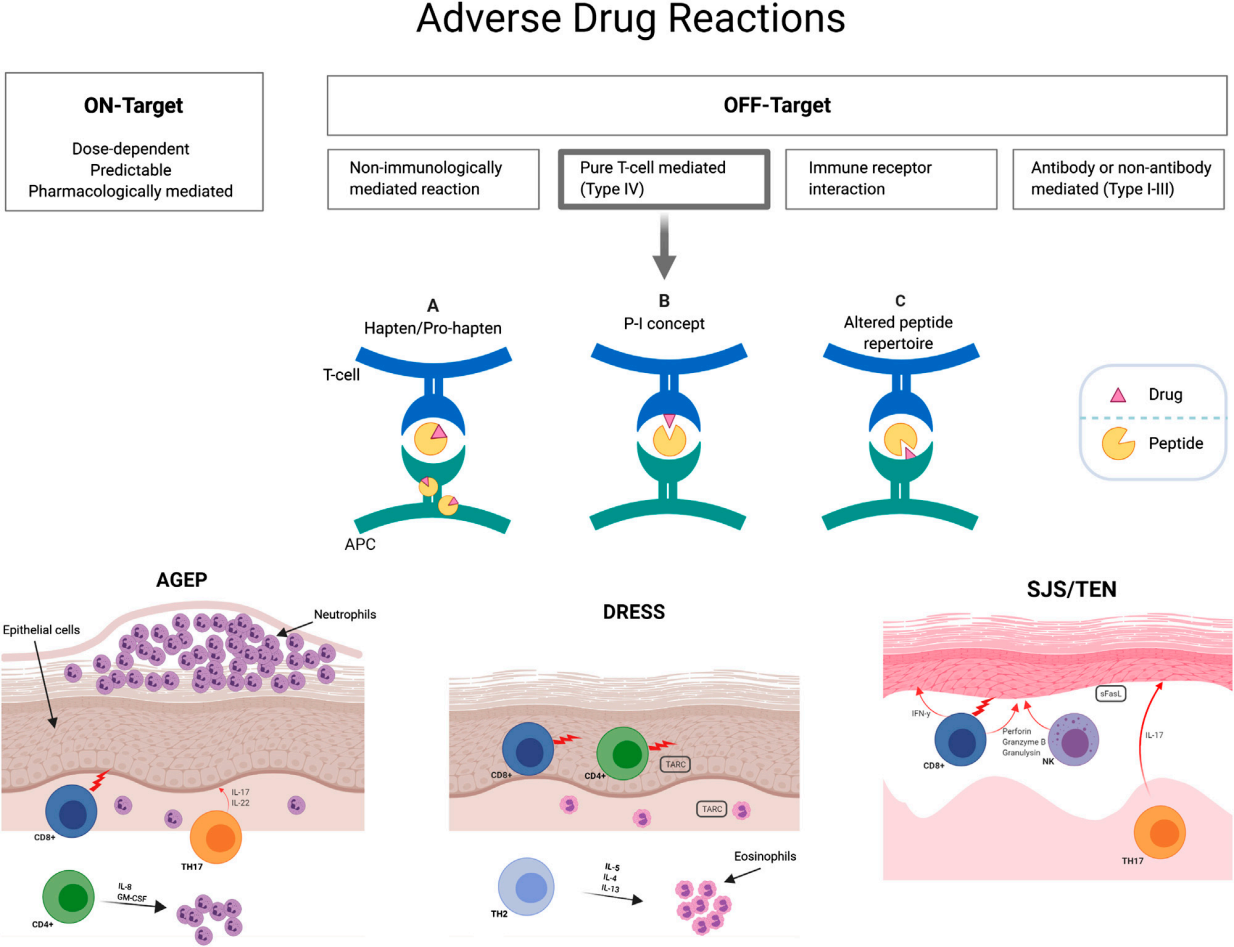
T-cell mediated delayed hypersensitivity mechanistic hypotheses. Three non-mutually exclusive hypotheses have been described to clarify drug triggered T-cell activation: **(A)** The hapten/prohapten model describes how an antigen (drug) that covalently binds to a self-peptide is intracellularly processed and then presented by MHC to T cells. **(B)** The p-i concept (pharmacological interaction with immune receptor) is based upon non-covalent binding of antigens to HLA or TCR without immune processing. **(C)** The altered repertoire model indicates that drugs can occupy positions in the peptide binding groove of the MHC, altering the binding cleft and the specificity of MHC binding. HLA, human leukocyte antigens; MHC, major histocompatibility complex; TCR, T-cell receptor.

With a better understanding of the pharmacogenomic and pathogenesis of drug reactions, newer classifications of adverse drug reactions that enhance our understanding of the drug hypersensitivity framework have been suggested. The on-target/off-target model categorizes adverse drug reactions by describing the interactions between drugs and their known targets for the desired pharmacological effect as well as the known or unknown mechanism for an off-target effect (White et al., 2015; Phillips, 2016). On-target reactions are generally non-immunologically mediated, dose-dependent and related to the primary pharmacologic mechanism of action of the drug. Off-target effects can relate to a number of known toxic, non-immunological and immunological mechanisms and can be subclassified in 1) dose dependent interactions with off-target receptors and pharmacological interactions such as non-IgE mediated mast cell activation or cellular toxicity and 2) drug allergy with immunological memory of variable duration such as delayed T-cell mediated reactions or IgE-mediated reactions (White et al., 2015). This is illustrated in Figure 1.

A well-established classification of historical relevance is the Gell and Coombs criteria of T-cell mediated hypersensitivity where type IVa is marked by T helper 1 (Th1) cells, macrophages and a secretion of IFN-γ, TNF-α, IL-18; type IVb by Th1 and other components such as B cells, IgE, IgG4, mast cells, eosinophils with a marked secretion of IL-4, IL-5 and IL-13; type IVc characterized by cytotoxic T cells that secrete granzyme, B perforin and granulysin and type IVd, where Th1/Th17 cells and neutrophils act through cytokine mediators such as GM-CSF, IL-8 and CXCL8 (Pichler and Hausmann, 2016). As the different phenotypes of delayed T-cell mediated reactions have different effector cells and cytokines, they have been portrayed under one of these subcategories with SJS/TEN probably related with CD8^+^ T-cell infiltrates (type IVc) and DRESS with a CD4^+^ dominant T-cell infiltration (type IVb) (Hari et al., 2001). The clear divergence in predominant cytokine signature between T-cell subsets provided indication for their detection to drive response categorization in each patient (see ELISpot section below).

This classification only partially accounts for underlying immunological mechanisms and does not explain the specific mechanism by which drugs may activate T cells. Three non-mutually exclusive hypotheses have been described to clarify drug triggered T-cell activation: 1) the p-i concept, 2) the hapten/pro-hapten model and 3) the altered peptide repertoire model (Figure 1). The pharmacological-interaction (p-i) model suggests that the offending drug can rapidly stimulate T cells by directly binding non-covalently to either T-cell receptor (TCR) or HLA (without antigen processing) (Pichler and Hausmann, 2016). This concept was proposed after observation that protein-unreactive drugs can stimulate T cells (Pichler and Watkins, 2014; White et al., 2015). In the hapten/pro-hapten model, novel antigens are generated from endogenous proteins that covalently bind the culprit drug or its metabolites, forming a neoantigen that then triggers T-cell response (Pichler and Hausmann, 2016). Haptens are small reactive molecule that become antigenic by covalent binding to high-molecular-weight autologous extracellular or cytoplasmic proteins. The resultant “haptenated” product undergoes presentation by APC on HLA molecules with subsequent activation of T cells. In this setting, re-exposure will generate rapid memory T-cell proliferation and inflammatory response. A classic example is the binding of penicillin metabolites to serum albumin (Padovan et al., 1996). Finally, in the altered peptide repertoire model, the causal drug occupies a position in the HLA peptide binding groove altering the binding cleft and the specificity of self-peptides able to bind to the HLA molecule (Illing et al., 2012). This model has only been established for abacavir hypersensitivity with the crystal structure of abacavir bound to peptide and HLA-B*57:01 having been described. It has hence been elucidated through this structure, peptide binding studies and peptide elution studies that abacavir binds non-covalently within the F pocket of the peptide-binding cleft of HLA-B*57:01 and alters the normal C9 peptide specificity from aromatic aliphatic amino acids, such as phenylalanine, to linear aliphatic amino acids, such as leucine, isoleucine and valine (Illing et al., 2012; Ostrov et al., 2012).

## Diagnostic Methods

### Drug Challenge

In the context of drug allergy, drug challenge in a patient with suspected drug-induced hypersensitivity remains the gold standard for determining tolerance (Aberer et al., 2003). For immediate reactions, such as IgE mediated reactions, a negative drug challenge has a 100% negative predictive value. However, in the case of a severe delayed reaction, re-challenge with a single dose of a drug may not reproduce the reaction and, hence, it has a lower sensitivity than a prolonged challenge (3–5 days), particularly with a remote reaction (Bousquet et al., 2008; Hjortlund et al., 2012). In particular settings such as childhood non-specific delayed mild exanthem associated with antibiotics in the context of a possible viral infection, there is increase evidence that direct oral challenge is a safe diagnostic tool (Mill et al., 2016; Trubiano et al., 2017a).

In addition, with high severity reactions, drug challenge carries an inherent risk and the benefit of re-challenge has to be carefully weighed against the risk of a serious reaction. In cases of severe cutaneous adverse reactions or severe organ involvement, challenges are contraindicated because of the risk of a life-threatening clinical reaction (Rive et al., 2013; Trubiano and Phillips, 2013). In this context, investigational tools have been developed to aid drug evaluation. *In vivo* testing such as PT and delayed IDT and *ex vivo* assays such as the LTT and ELISpot have been described for various drugs and phenotypes but lack international validation. Combining *in vivo* and *ex vivo* methods in delayed hypersensitivity reactions can increase the diagnostic yield, although this has been shown in only small cohort studies (Trubiano et al., 2018).

### Skin Testing

*In vivo* testing (PT and delayed IDT) is usually performed to the implicated drug(s) at least 4–6 weeks after delayed hypersensitivity resolution at the recommended non-irritating concentrations (Phillips et al., 2019).

### Patch Testing

The main types of reactions where PT is used with high specificity are MPE, AGEP, DRESS, SJS/TEN and FDE (Ozkaya-Bayazit et al., 1999; Barbaud et al., 2001; Barbaud et al., 2013). The sensitivity of this investigational tool varies depending on the clinical setting, the causal drug, the drug concentrations used and the phenotype with typical figures for AGEP at 58–64% (Wolkenstein et al., 1996; Barbaud et al., 2013), DRESS between 32 and 80% (Barbaud et al., 2013; Barbaud, 2014) and SJS/TEN, 9–24% (Wolkenstein et al., 1996; Barbaud et al., 2013). Drugs like antiepileptics, contrast media, beta-lactams, tetrazepam and pristinamycin increase the sensitivity of PT (Johansen et al., 2015), while allopurinol or its active metabolite, oxypurinol, appear to never provide clinical utility.

The testing should be performed at least one month after the resolution of the reaction or after discontinuation of oral steroids, as immunosuppressants can decrease T-cell mediated immunity, and preferably during the first year after the reaction. The European Network on drug allergy (ENDA) and the European Academy of Allergy and Clinical Immunology (EAACI) recommend timing between 3 weeks and 3 months and describe drug concentrations between 5 and 30% with most antimicrobials diluted at 20% (Brockow et al., 2002) or 30% (Barbaud et al., 2001) in petrolatum vehicle and the retained vehicle alone as negative control (Barbaud et al., 2001; Brockow et al., 2013). For DRESS, patch testing may be further delayed because of the concomitant dosing of topical or systemic steroids or other immunosuppressants and to avoid confusion with DRESS relapse. Available literature suggests that the yield from patch testing for SJS/TEN is in general low but dependent on the drug and class of drugs. Sensitivities will vary from 0% for allopurinol to >50% for aromatic antiepileptic drugs such as carbamazepine (Konvinse et al., 2016).

The two forms of PT described are the extemporaneous, involving the local preparation of the PT by the pharmacy or the drug allergy staff with commercially available drugs and petrolatum or water, and the conventional PT implying use of a limited number of ready-to-use commercialized PT products at 10% concentration in petrolatum (Chemotechnique, Sweden). In a retrospective study, 21/75 (23.3%) patients with MPE, FDE, AGEP, DRESS, SJS/TEN tested simultaneously with both methods had positive results, indicating that both methods are as valuable and reliable (Assier et al., 2017). PT is usually applied in the upper back regions for practical reasons with the exception of FDE in which the PT is applied on the region of the previous reaction. The International Contact Dermatitis Research Group have published an interpretation score for the patch test reactions (Table 2) (Barbaud et al., 2001).

**TABLE 2:** Score—Interpretation of patch testing reactions.

Score	Interpretation
−	Negative reaction
? or +/−	Doubtful reaction, faint erythema
+	Weak reaction, erythema, slight infiltration
++	Strong reaction, erythema, infiltration, papules or vesicles (bullae)
	Reaction may extend beyond the margins of the patch
+++	Extreme, bullous, ulcerative
IR	Irritant reaction: Follicular, pustular, bullous or necrotic
NT	Not tested

In a large multi-center patch testing cohort, only one patient (1/134) presented a relapse of his skin condition (AGEP) following patch testing (Barbaud et al., 2013) indicating that this diagnostic method carries low morbidity. In a retrospective review including 826 patients, PT showed promising results for drug challenge outcomes with 82.3% (14/17) with positive PT having a positive challenge and 90.4% (207/229) patients with negative PT presenting no reaction to challenge (Lammintausta and Kortekangas-Savolainen, 2005).

PT is a quick and safe investigational method clinically relevant when testing is conclusive, a negative PT not excluding the possibility that the drug is causal. There is need to re-challenge negative testing in less severe clinical phenotypes. This method should be homogenized, as to resolve current inconsistencies, by comparing the outcomes in large multicenter studies, determining concentration thresholds and avoiding false negative and false positive results.

### Intradermal Testing

Intradermal testing is done on the volar aspect of the forearm with 0.02–0.05 ml of antibiotic reagent or normal 0.9% serum saline (negative control) (Empedrad et al., 2003a; Brockow et al., 2013). The use of IDT is limited to drugs available in liquid sterile formulations. The positive control normally used is a skin prick test with histamine 10 mg/ml (Heinzerling et al., 2013). In terms of drug concentrations, expert consensus advises the use of the highest non-irritating concentration described for immediate reactions (Phillips et al., 2019). However, recent work for drugs with non-IgE mast cell activation determined that higher concentrations that might initially be irritating are needed for improved sensitivity (i.e., ciprofloxacin, vancomycin) (Brockow et al., 2002; Brockow et al., 2013; Konvinse et al., 2016). An IDT result is considered positive when the dermal induration and erythema at the injection site exceeded 5 mm from baseline (Empedrad et al., 2003b; Brockow et al., 2013). Delayed reading is performed at 24, 48 h and up to 1 week (Empedrad et al., 2003b; Brockow et al., 2013). IDT with delayed reading has been described in reactions such as MPE, AGEP and DRESS with potential risk in SJS/TEN and unknown utility in FDE (Table 3). This investigational tool was previously considered potentially harmful in SCAR phenotypes but actually few reports describe severe systemic reactions following IDT (Makris et al., 2010; Sala Cunill et al., 2011; Syrigou et al., 2016; Watts, 2017). For SJS/TEN, based on the current available literature, the benefit of IDT does not outweigh the risk. For DRESS, it is recommended that testing generally be deferred 6 months following the acute reaction.

**TABLE 3:** Role of diagnostic/screening tests in delayed drug hypersensitivity reactions.

	*In vivo*			*Ex vivo*		
Clinical diagnosis	Patch testing	Delayed IDT	Oral challenge	LTT	ELISpot	HLA
MPE	Yes ♣	Yes ♣	Yes	No	No	No
AGEP	Yes	Yes	No	Equivocal	Equivocal	No
DRESS/DIHS	Yes	Yes	No	Yes	Yes	Yes ψ
SJS/TEN	Yes	No	No	Yes	Yes	Yes ψ
FDE	Yes ω	No	Equivocal	No	No	No
SDRIFE	Yes ω	Equivocal	Equivocal	No	No	No

AGEP, acute generalized exanthematous pustulosis; DIHS, Drug-induced Hypersensitivity syndrome; DRESS, Drug reaction with eosinophilia and systemic symptoms; ELISpot, enzyme-linked immunospot; FDE, fixed drug eruption; HLA, human leukocyte antigen; LTT, Lymphocyte transformation test; SDRIFE, symmetrical drug-related intertriginous and flexural exanthema, SJS/TEN, Stevens-Johnson syndrome/toxic epidermal necrolysis. ♣ As the sensitivity for PT and IDT is poor, drug challenge of the implicated drug can be considered. PT/IDT may give information on cross-reactivity ω PT should be applied on the region of the previous reaction ψ HLA screening is not routinely used globally in clinical practice. Please refer to Table 4 for details.

In terms of cross-reactivity between beta-lactams in the context of delayed hypersensitivities, 18.7–31.2% of the patients tested presented a reaction to amino-penicillins and amino-cephalosporins (Dash, 1975) predicted by the presence of shared R1 and R2 side chains (Buonomo et al., 2014; Romano et al., 2016). Also, in patients with a delayed penicillin type reaction, delayed IDT to beta-lactams has allowed to confirm tolerance to cephalosporins (Picard et al., 2019; Trubiano et al., 2020), carbapenems (Gaeta et al., 2015; Picard et al., 2019) and monobactams (Buonomo et al., 2011). Other classes of interest are currently being studies with no evidence of cross-reactivity such as glycopeptides (Empedrad et al., 2003a), antibiotic and non-antibiotic sulfonamides (Empedrad et al., 2003a; Lammintausta and Kortekangas-Savolainen, 2005), drugs in the rifampin class (Lammintausta and Kortekangas-Savolainen, 2005) and aromatic and non-aromatic anticonvulsants (Heinzerling et al., 2013).

In the setting of a severe delayed reaction, PT is related to lower adverse reactions but IDT has been described as more sensitive in non-SJS/TEN reactions (Osawa et al., 1990; Barbaud et al., 2001; Cabanas et al., 2014) while some recommendations only suggest proceeding to IDT after negative PT (Brockow et al., 2002). In a cohort study of 21 patients with delayed reactions to penicillin and 30 controls with no allergic history, no false positives were reported and 20/21 were positive for IDT compared to 18/21 for patch testing (Torres et al., 2004).

Widespread implementation of IDT for delayed hypersensitivities still carries some barriers such as the lack of available sterile preparation for all drugs, generally low negative predictive value (NPV) and limited data in some reactions.

### *Ex Vivo* Diagnostic Tools

**FIGURE 2:**
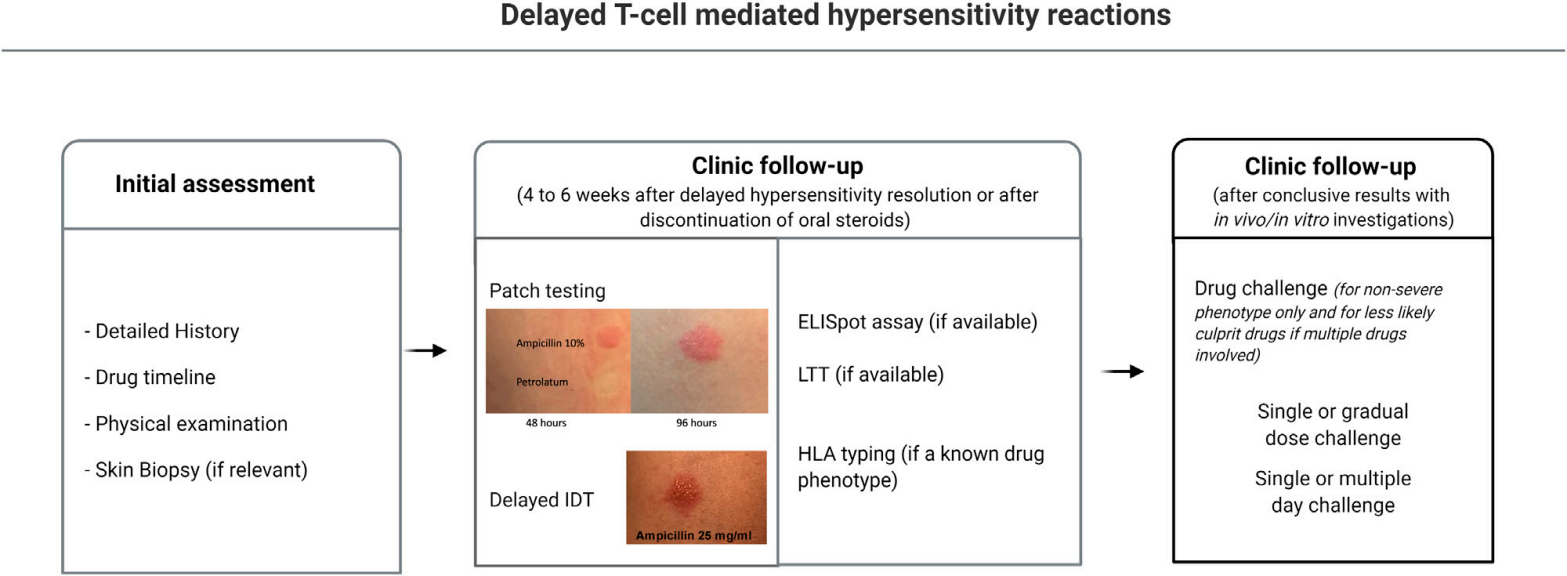
Diagnostic approach to delayed hypersensitivity reactions. 1) Clinical history and identification of possible causal drugs; 2) Patch testing; 3) Intradermal testing reading after 24–48 h; 4) *Ex-vivo* testing (if available): ELISpot, LTT, HLA allele testing; 5) Single or prolonged drug challenge if conclusive results. ELISpot, enzyme linked ImmunoSpot; HLA, human leukocyte antigens; LTT, lymphocyte transformation test.

*In vitro/ex vivo* diagnostics, such as the LTT and the ELISpot assay, while having the advantage of carrying no risk of drug re-exposure for the patient, are not available for routine diagnostic use in most centers. A practical management approach for delayed T-cell mediated hypersensitivity reactions is illustrated in Figure 2.

### Lymphocyte Transformation Test

LTT has been extensively studied as a diagnostic method for delayed hypersensitivity reactions. Lymphocytes are isolated from the patient’s peripheral blood mononuclear cells (PBMC) and cultured with pharmacological concentrations of the suspected drugs for 5–7 days. LTT responses are measured by the stimulation index (SI, average proliferation of drug-exposed cultures/average proliferation of negative control cultures), with typically an SI > 2+ confirming response, which is calculated based on the radioactive thymidine (H-thymidine) uptake, a marker directly proportional to the degree of T-cell proliferation in response to a drug antigen (Pichler and Tilch, 2004). This enhanced response is interpreted as a T-cell sensitization and has produced positive responses in different clinical settings and with various implicated drugs (Rive et al., 2013). However, one might keep in mind that lymphocyte stimulation can occur not only by immunological mechanisms but also pharmacological ones and some drugs may cause false positive results as was observed in some patients that presented positive responses to drugs they had tolerated (Pichler and Tilch, 2004).

The reported sensitivity of LTT in delayed hypersensitivity reactions ranges from 27% (Porebski et al., 2013) to 74% (Nyfeler and Pichler, 1997) and specificity was quoted as 85% (Nyfeler and Pichler, 1997; Rozieres et al., 2009) to 100% (Porebski et al., 2013; Porebski et al., 2015). Putting aside the demanding and time-consuming laboratory manipulations and the use of radioactivity and specialist equipment, the LTT can be an interesting support in drug hypersensitivity diagnosis but is still only used as a research tool (Pichler and Tilch, 2004; Nagao-Dias et al., 2009). The largest study describes LTT in 923 patients with suspected hypersensitivity among which only 100 patients had a confirmed drug hypersensitivity reaction and 58/78 penicillin allergy labeled patients presented a positive LTT (Picard et al., 2019). In the last 10 years, aside from case reports or small cases series (Kim et al., 2013; Cabanas et al., 2014; Dias de Castro et al., 2015; Tomida et al., 2016), very few studies have focused solely on the LTT method for diagnosis.

### Enzyme Linked ImmunoSpot

**FIGURE 3:**
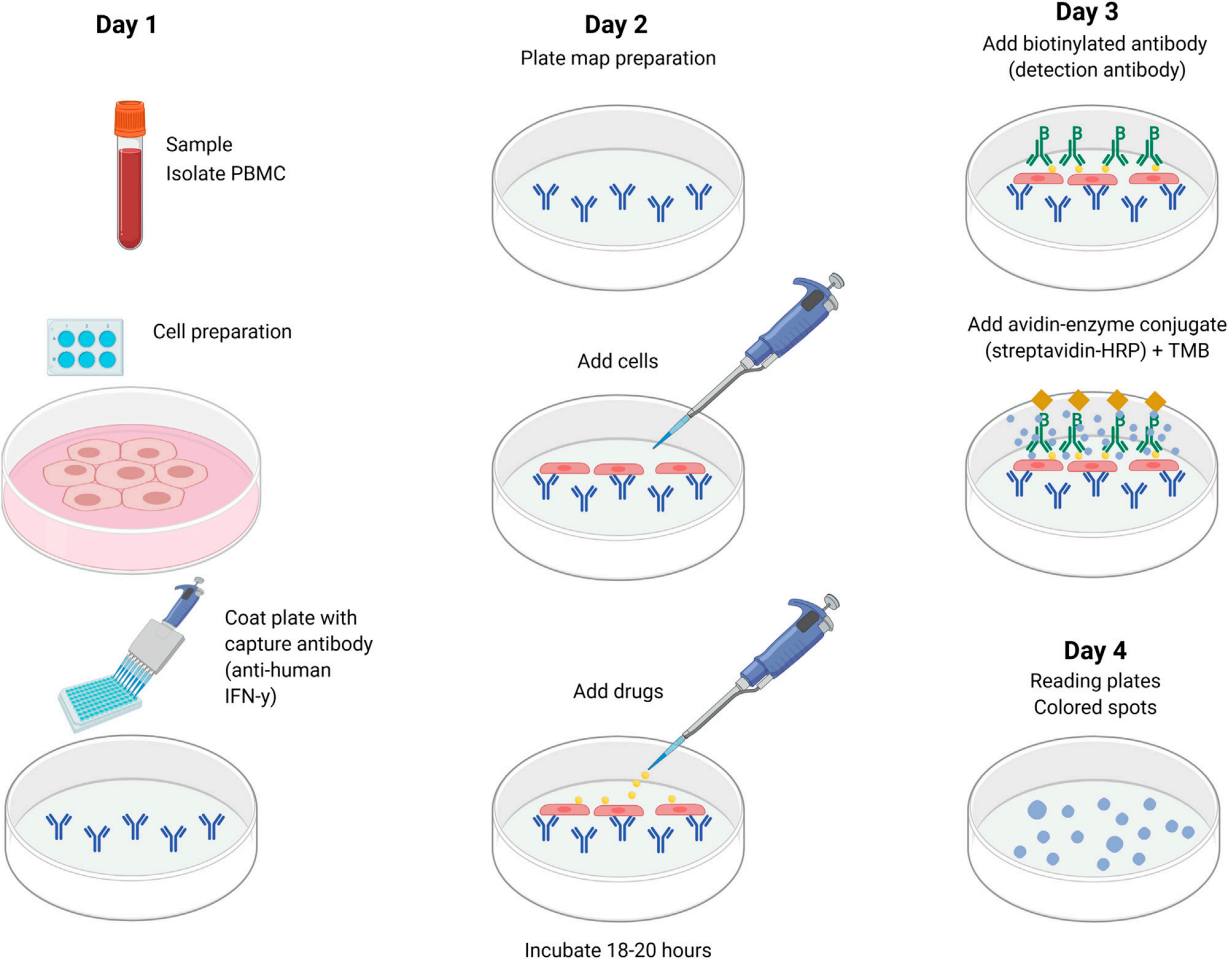
Detection of cytokine secretion using ELISpot. Cytokine-specific coating antibody is added and incubated overnight at 4°C. The plate is washed and PBMCs and drug(s) are added and incubated for 18–20 h at 37°C. The following day, the plate is washed again and biotin-conjugated detection antibody is added and incubated for 2 h at room temperature. After another wash, streptavidin-bound enzyme is added and incubated for 1 h at room temperature. After the last wash, substrate is added (BCIP-NBT or TMB). Spot development is monitored for ∼15 min (in dark). The plate is washed and left to dry overnight for a final reading on the fourth day. BCIP-NBT, 5-bromo-4-chloro-3-indolyl phosphate (BCIP)/nitro blue tetrazolium (NBT); ELISpot, enzyme linked ImmunoSpot; PBMC, peripheral blood mononuclear cells; TMB, tetramethyl benzidine.

The ELISpot technique quantifies the secretion and activation of drug-specific cells by determining the number of spot-forming units (SFU) or spot-forming cells (SFC) that release cytokine markers or cytolytic molecules after the patient’s PBMC is activated with the suspected drug(s) (Figure 3). The patient’s cells are added to a 96-well plate coated with specific anti-cytokine antibody depending on the expected measured T-cell response. In drug-induced delayed hypersensitivity, interferon gamma (IFN-γ), a key Th1-type cytokine, is released from activated T cells, while granzyme B (GrB), a serine protease, is released from cytoplasmic granules within cytotoxic T cells and natural killer cells. Anti-CD3 antibodies, a mix of viral peptides CEF (cytomegalovirus (CMV), Epstein-Bar Virus (EBV) and influenza (FLU)) and tetanus toxoid can be used as positive controls as they stimulate INF-γ release from CD8^+^ T cells. The background immunological activation can be assessed with negative controls (cells and media). Cytokine secretion is captured by the anti-cytokine antibodies in the next 24–48 h with detection antibody and enzyme substrate being added just before reading the plate. The SFU representing cells that secrete cytokines are then identified and counted. As the incubation time is shorter than for LTT and T-cell activation occurs after 48–72 h, this could be a promising technique. However, one must consider the often-diverse response between replicates and the researcher intensive laboratory manipulations.

In a recent study, the sensitivity of this technique in patients with SCAR was 52%, 10/19 patients presenting a positive IFN-γ ELISpot (>50 SFU/10^6^), with a specificity of 100% (Trubiano et al., 2018). The GrB ELISpot has a lower sensitivity (33%; 5/15 positive patients (>20 SFU/well) (Porebski et al., 2013) up to 55%; 13/23 positive patients (>0 SFU) (Porebski et al., 2015)) and similar specificity. However, when compared to LTT, ELISpot seems to have a better sensitivity (Rozieres et al., 2009). Depending on the positive ELISpot assay definition, the number of confirmed cases varies with several studies considering the unique presence of SFU as sufficient for a positive test (Khalil et al., 2008; Porebski et al., 2015; Castagna et al., 2018). A recent study from our group reported 5/12 positive ELISpot among SCAR patients with a 50 SFU/10^6^ cut-off (Trubiano et al., 2020). However, in a cohort of 22 patients with amoxicillin MPE, the sensitivity of the IFN-γ ELISpot was 91% (15/22) when the cut-off used was more than 30 SFU/10^6^ (Rozieres et al., 2009). Using this same reference value, the sensitivity was 87.5% (7/8) in a study involving eight patients with hypersensitivity to piperacillin (El-Ghaiesh et al., 2012). Finally, some authors determine a positive value based solely on the SFU detection level in controls (Suthumchai et al., 2018). Because of the current controversy in the literature, our definition of a positive response is equal or greater than 50 spot-forming unit (SFU)/million cells after background (unstimulated control) (Keane et al., 2012).

As discussed, LTT and ELISpot do not have a good sensitivity especially when the blood is collected during the acute reaction. One hypothesis is that the reactive cells are not found in the circulation or that overstimulated lymphocytes could be exhausted. Thus, a cytokine or cytolytic marker panel could help delineate the implicated mediators. While alternative cell viability and proliferative assays have been developed in recent years including several variants of the MTT and carboxyfluorescein succinimidyl ester (CFSE) staining assays, these have not widely been applied for diagnostic investigation due to issues surrounding potential drug-inhibition of metabolism-dependant colorimetric conversion and flow cytometer access, and difficulties in staining, respectively.

### Biomarkers in Adverse Drug Reactions

As the most severe reactions but also those with the most varied clinical presentations are SJS/TEN and DRESS/DiHS, research efforts have been concentrated to develop new biomarkers with a particular interest in cytokines and chemokines released from activated T cells.

Studies on cytokine measurements after clinical drug challenge in patients with the generalized form of FDE clinically and histologically mimicking SJS/TEN have reported an initial increase in serum TNF-α and IL-8 followed by elevation in IFN-γ, IL-6 and IL-10 levels (Kauppinen, 1991; Correia et al., 2002; Shiohara et al., 2015). Similarly, dosage of levels for multiple cytokines/chemokines in order to identify essential markers has also been attempted with studies identifying a significant increase in IL-6 and interferon gamma-produced protein 10 (IP-10) in SJS/TEN and DRESS as well as IL-16 in FDE, SJS and DRESS but not TEN (Shiohara et al., 2015). These authors go to recommend the use of IL-6 and IL-10 as diagnostic and predictive tools in monitoring adverse drug reactions (Shiohara et al., 2015). On a cautionary note, these markers may be elevated in other conditions such as acute infection and sepsis.

Further, serum soluble Fas-ligand (sFasL) levels (Posadas et al., 2002; Abe et al., 2003; Murata et al., 2008), granulysin (Chung et al., 2008; Porebski et al., 2013; Cho et al., 2014; Chung et al., 2015; Weinborn et al., 2016), IL-15 (Su et al., 2017), CD137 (Trubiano et al., 2017b) and the proapoptotic factor galectin-7 (Hama et al., 2019) have been described in the pathological processes of SJS/TEN with sFasL and galectin-7 being considered as biomarkers able to predict TEN progression but not SJS (Shiohara et al., 2015; Hama et al., 2019) and granulysin serum levels correlating with disease severity and mortality (Chung et al., 2008; Chung et al., 2015). In DRESS/DiHS, several markers were reported as indicators of disease progression and activity such as the serum thymus and activation-regulated chemokine (TARC) (Ogawa et al., 2013; Komatsu-Fujii et al., 2017; Komatsu-Fujii et al., 2018) and granulysin (Saito et al., 2012). Other markers such IL-2, IL-4, IL-5, IL-13, IFN-γ and granzyme-B have been described in T-cell drug hypersensitivity (Lochmatter et al., 2009; Zawodniak et al., 2010; Polak et al., 2013). Measurement of these markers was reported using the ELISpot, intracellular cytokine staining, ELISA, rapid immunochromatographic tests (Su et al., 2016), plex bead-based immunoassay kits (Lochmatter et al., 2009) and flow cytometry (Porebski et al., 2013). Controversial markers are also important to underline such as IL-17 with some studies reporting a negative correlation with adverse drug reactions (Shiohara et al., 2015) while others described an increase of this cytokine in SJS/TEN (Teraki et al., 2013). Similarly, procalcitonin has been described as a marker for bacterial infection that could benefit the differential diagnostic that includes delayed hypersensitivity (Yoon et al., 2013).

In the early stages of severe delayed hypersensitivity disease, laboratory tests that can be used in clinical routine are needed to predict disease progression and to monitor treatment responses.

### High-Resolution Human Leukocyte Antigen Class I and II Typing

The association between particular class I HLA alleles and specific phenotypes such as allopurinol SJS/TEN and DRESS (HLA-B*58:01), carbamazepine SJS/TEN (HLA-B*15:02) and abacavir hypersensitivity reaction and flucloxacillin drug-induced liver injury (HLA*57:01) has allowed a better understanding of the immunopathogenesis of severe T-cell mediated delayed hypersensitivity reactions and the implementation of guidelines and screening programs in the case of HLA-B*57:01 and abacavir and HLA-B*15:02 and carbamazepine in Southeast Asian populations in particular (Table 4).

**TABLE 4:** HLA associations in SCAR and DILI with possible clinical implications.

Reference	Reaction type	Drug	HLA	Ethnicity	Screening	NPV (%)	PPV (%)	NNT
(Konvinse et al. (2019))	DRESS	Vancomycin	A*32:01	European ancestry (6.8%)	Pre-emptive♣	99.99	0.51	75
				African American (4%)				
				Southeast Asian (<1.5%)				
(Daly et al., (2009))	DILI	Flucloxacillin	B*57:01	European ancestry (5–8%)	None	99.99	0.14	13,819
				African American (2.5%)				
				African/Asia (<1%)				
(Mallal et al. (2002))	AB HS	Abacavir	B*57:01	Caucasian (5–8%)	HIV positive patients	100	55	13
(Hung et al. (2005))	SJS/TEN	Allopurinol	B*58:01	Han Chinese (9–11%)	None	100	3	250
	DRESS			Caucasian (1–6%)				
(Zhang et al. (2013))	SJS/TEN	Carbamazepine	B*15:02ψ	Han Chinese (10–15%)	Routine in southeast Asian countries	100	3	1,000
(Zhang et al. (2013))	DRESS	Dapsone	B*13:01	Papuans/Australian aborigines (28%)	Leprosy patients in countries with increased prevalence	99.8	7.8	84
				Chinese (2–20%)				
				Japanese (1.5%)				
				Indian (1–12%)				

AB HS, abacavir hypersensitivity syndrome; DILI, drug-induced liver injury; DRESS, drug reaction with eosinophilia and systemic symptoms; HIV, human immunodeficiency virus; NNT, numbers needed to test (to prevent one case); NPV, negative predictive value; PPV, positive predictive value; SJS/TEN, SJS/TEN, Stevens-Johnson syndrome/toxic epidermal necrolysis. ♣ HLA-A*32:01 testing could have a role in determining the culprit drug (vancomycin) when multiple drugs are implicated in a delayed hypersensitivity reaction.ψ Other described alleles: HLA-B*15:21, HLA-B*15:11, and HLA-B*15:18.

**FIGURE 4:**
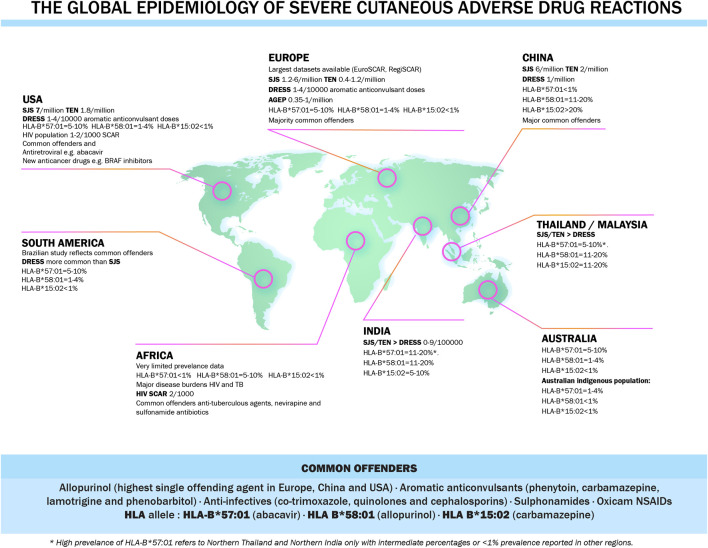
Global epidemiology of severe cutaneous adverse drug reactions.

DNA from patients with drug reactions can be obtained by a routine blood draw and extracted from whole blood or extracted from saliva collected into, for instance, a gene collection kit. DNA can then be used to perform high resolution HLA class I and II typing with next generation sequencing methods. To facilitate HLA testing with rapid turnaround times, cost-effective single allele assays have been developed for many class I HLA alleles such as HLA-B*57:01, HLA-B*15:02 and HLA-A*32:01 with parallel allele specific quality assurance programs which was crucial for the widespread global implementation of HLA-B*57:01 screening programs. HLA genes encode cell-surface protein receptors that present antigenic peptides to T cells. Class I MHC molecules (HLA-A, B and C) are expressed on most nucleated cells and are responsible for presenting peptides to CD8^+^ cytotoxic T lymphocytes. Class II MHC molecules (HLA-DP, DQ and DR) are expressed only on antigen presenting cells (B cells, macrophages and dendritic cells) and stimulate CD4^+^ helper T lymphocytes. The association between HLA and disease confers explanations on disease susceptibility with HLA polymorphisms playing a crucial role in T-cell repertoire and auto-reactive T cells, immune system presentation, recognition and antigen processing and the adaptability of the immune system (Shiina et al., 2004). Also, HLA allele have a different prevalence in different ethnic groups and this might explain the increased drug reactions in specific populations (Rive et al., 2013). The global epidemiology of severe cutaneous adverse drug reactions is illustrated in Figure 4.

Currently, screening for HLA-B*57:01 prior to abacavir prescription is the standard of care in HIV clinical practice across the developed world. When screening occurs and is acted upon, it eliminates abacavir hypersensitivity (Mallal et al., 2008). Another example is screening for HLA-B*15:02 before initiating treatment with carbamazepine to avert SJS/TEN in some Southeastern Asian countries with increased prevalence. The xanthine oxidase inhibitor, allopurinol, was also associated with SJS/TEN and DRESS and HLA-B*58:01genotyping in Han Chinese showed 100% NPV and 3% PPV (Hung et al., 2005), however it has incomplete negative predictive value in European and African populations where 50–60% of patients with allopurinol DRESS/SJS/TEN do not carry HLA-B*58:01 (Lonjou et al., 2006).

Many of the described HLA alleles associations have a close to 100% negative predicting value, however this is highly dependent on the prevalence of the HLA allele in the population and the risk allele(s) in different populations. For instance, for allopurinol SJS/TEN and DRESS and HLA-B*58:01, it has almost a 100% NPV in Southeast Asian population however explains only 50–60% of allopurinol DRESS/SJS/TEN in European and African populations. The number needed to test to prevent one case of disease is thus population specific. However, as the prevalence of these diseases in the general population is reduced, more targeted populations could benefit from screening.

### Recommendations

- Patch testing and intradermal testing can be used in the clinical setting for specific clinical diagnosis of T-mediated delayed hypersensitivity reactions while appreciating that preparations and drug concentration should be standardized to optimize their use.
- While skin testing (patch testing and intradermal testing) has a high drug specificity, both have phenotype and drug dependent sensitivity and incomplete NPV and, in the setting of severe delayed drug reaction, clinical history is the main determinant of drug safety that guides the decision for drug challenge or future drug use.
- Different non-irritating drug concentrations have been described for intradermal and patch testing. However, global consensus is lacking and clinicians are encouraged to follow the most recent drug allergy guidelines.
- Intradermal testing can be safety performed for non-SJS/TEN delayed reactions. Patch testing is the initial *in vivo* investigational tool that can be used for severe delayed reactions such as SJS/TEN.
- *Ex vivo* tools such as the lymphocyte transformation test and the enzyme-linked ImmunoSpot assay are currently not available for routine clinical practice and are used solely in specialized center. Collaborating with such a center will not only improve patient care but could benefit research in this field.
- In the early stages of severe delayed hypersensitivity disease, laboratory tests that can be used in clinical routine are needed to predict disease progression and to monitor treatment responses. There are currently no tests that should be order on a routine basis.
- Strong HLA associations with delayed T-cell mediated hypersensitivity reactions have enlightened our understanding of their immunopathogenesis and, in combination with availability of cost-effective single HLA testing, have provided a pathway for pre-prescription screening strategies. In the future, HLA testing may be increasingly relevant for pre-emptive testing and diagnosis.
- There is currently no diagnostic tool that offers a 100% NPV for the delayed hypersensitivity reactions and any decision to reintroduce a drug in the treatment setting should weigh the risk benefit ratio.

## Conclusion

Identifying culprit drugs implicated in delayed T-cell mediated hypersensitivity with the use of exemplary clinical phenotyping, clinical drug causality assessment and adjunctive *in vivo* and *ex vivo* testing including HLA-typing is increasingly useful to guide safe and optimal future treatment.

## Author Contributions

All authors listed have made a substantial, direct, and intellectual contribution to the work and approved it for publication.

## Funding

EP receives support from 1P50GM115305, R21AI139021, 1 R01 HG010863, and 1R01AI152183.

## Conflict of Interest

The authors declare that the research was conducted in the absence of any commercial or financial relationships that could be construed as a potential conflict of interest.

## Glossary

ADR: adverse drug reactions
AGEP: acute generalized exanthematous pustulosis
ALDEN: algorithm of drug causality for epidermal necrolysis
CEF: cytomegalovirus (CMV) Epstein-Bar Virus (EBV) and influenza (FLU)
CFSE: carboxyfluorescein succinimidyl ester
DILI: drug induced liver injury
DRESS: drug reaction with eosinophilia and systemic symptoms
ELISpot: enzyme linked ImmunoSpot
FBS: fetal bovine serum
FDE: fixed drug eruption
GrB: granzyme B
HLA: human leukocyte antigens
IFN-γ: Interferon gamma
IP-10: interferon gamma-produced protein 10
LTT: lymphocyte transformation test
MHC: major histocompatibility complex
MPE: maculopapular exanthema
MTT: (3-(4,5-dimethylthiazol-2-yl)-2,5-diphenyltetrazolium bromide)
NPV: negative predictive value
PBMC: peripheral blood mononuclear cells
PPV: positive predictive value
SCAR: severe cutaneous adverse reactions
SCORTEN: SCORe of toxic epidermal necrosis
SDRIFE: symmetrical drug-related intertriginous and flexural exanthema
sFasL: soluble Fas-ligand
SFC: spot-forming cells
SI: stimulation index
SJS: Stevens-Johnson syndrome
TARC: thymus and activation-regulated chemokine
TCR: T-cell receptor
TEN: toxic epidermal necrolysis

## References

[B1] Abe R. Shimizu T. Shibaki A. Nakamura H. Watanabe H. Shimizu H. (2003). Toxic epidermal necrolysis and Stevens-Johnson syndrome are induced by soluble Fas ligand. Am. J. Pathol. 162 (5), 1515–1520. 10.1016/S0002-9440(10)64284-8 12707034 PMC1851208

[B2] Aberer W. Bircher A. Romano A. Blanca M. Campi P. Fernandez J. (2003). Drug provocation testing in the diagnosis of drug hypersensitivity reactions: general considerations. Allergy. 58 (9), 854–863. 10.1034/j.1398-9995.2003.00279.x 12911412

[B3] Assier H. Valeyrie-Allanore L. Gener G. Verlinde Carvalh M. Chosidow O. Wolkenstein P. (2017). Patch testing in non-immediate cutaneous adverse drug reactions: value of extemporaneous patch tests. Contact Dermatitis. 77 (5), 297–302. 10.1111/cod.12842 28730661

[B4] Barbaud A. Gonçalo M. Bruynzeel D. Bircher A., European Society of Contact Dermatitis (2001). Guidelines for performing skin tests with drugs in the investigation of cutaneous adverse drug reactions. Contact Dermatitis. 45 (6), 321–328. 10.1034/j.1600-0536.2001.450601.x 11846746

[B5] Barbaud A. Collet E. Milpied B. Assier H. Staumont D. Avenel-Audran M. (2013). A multicentre study to determine the value and safety of drug patch tests for the three main classes of severe cutaneous adverse drug reactions. Br. J. Dermatol. 168 (3), 555–562. 10.1111/bjd.12125 23136927

[B6] Barbaud A. (2014). Skin testing and patch testing in non-IgE-mediated drug allergy. Curr. Allergy Asthma Rep. 14 (6), 442 10.1007/s11882-014-0442-8 24740692

[B7] Bastuji-Garin S. Fouchard N. Bertocchi M. Roujeau J. C. Revuz J. Wolkenstein P. (2000). SCORTEN: a severity-of-illness score for toxic epidermal necrolysis. J. Invest. Dermatol. 115 (2), 149–153. 10.1046/j.1523-1747.2000.00061.x 10951229

[B8] Bousquet P. J. Pipet A. Bousquet-Rouanet L. Demoly P. (2008). Oral challenges are needed in the diagnosis of beta-lactam hypersensitivity. Clin. Exp. Allergy. 38 (1), 185–190. 10.1111/j.1365-2222.2007.02867.x 17976216

[B9] Brockow K. Romano A. Blanca M. Ring J. Pichler W. Demoly P. (2002). General considerations for skin test procedures in the diagnosis of drug hypersensitivity. Allergy. 57 (1), 45–51. 10.1046/j.0105-4538.2001.00001.x-i8 11991289

[B10] Brockow K. Garvey L. H. Aberer W. Atanaskovic-Markovic M. Barbaud A Bilo M. B. (2013). Skin test concentrations for systemically administered drugs–an ENDA/EAACI Drug Allergy Interest Group position paper. Allergy. 68 (6), 702–712. 10.1111/all.12142 23617635

[B11] Buonomo A. Nucera E. De Pasquale T. Pecora V. Lombardo C. Sabato V. (2011). Tolerability of aztreonam in patients with cell-mediated allergy to β-lactams. Int. Arch. Allergy Immunol. 155 (2), 155–159. 10.1159/000318844 21196760

[B12] Buonomo A. Nucera E. Pecora V. Rizzi A. Aruanno A. Pascolini L. (2014). Cross-reactivity and tolerability of cephalosporins in patients with cell-mediated allergy to penicillins. J Investig. Allergol. Clin. Immunol. 24 (5), 331–337. 25345303

[B13] Cabanas R. Calderon O. Ramirez E. Fiandor A. Prior N. Caballero T. (2014). Piperacillin-induced DRESS: distinguishing features observed in a clinical and allergy study of 8 patients. J Investig. Allergol. Clin. Immunol. 24 (6), 425–430. . 25668894

[B14] Castagna J. Nosbaum A. Vial T. Rozieres A. Hacard F. Vocanson M. (2018). Drug-induced aseptic meningitis: a possible T-cell-mediated hypersensitivity. J. Allergy Clin. Immunol. Pract. 6 (4), 1409–1411. 10.1016/j.jaip.2017.11.034 29310902

[B15] Cho Y. T. Lin J. W. Chen Y. C. Chang C. Y. Hsiao C. H. Chung W. H. (2014). Generalized bullous fixed drug eruption is distinct from Stevens-Johnson syndrome/toxic epidermal necrolysis by immunohistopathological features. J. Am. Acad. Dermatol. 70 (3), 539–548. 10.1016/j.jaad.2013.11.015 24388722

[B16] Chung W. H. Hung S. I. Yang J. Y. Su S. C. Huang S. P. Wei C. Y. (2008). Granulysin is a key mediator for disseminated keratinocyte death in Stevens-Johnson syndrome and toxic epidermal necrolysis. Nat. Med. 14 (12), 1343–1350. 10.1038/nm.1884 19029983

[B17] Chung W. H. Chang W. Stocker S. L. Juo C. G. Graham G. G. Lee M. H. (2015). Insights into the poor prognosis of allopurinol-induced severe cutaneous adverse reactions: the impact of renal insufficiency, high plasma levels of oxypurinol and granulysin. Ann. Rheum. Dis. 74 (12), 2157–2164. 10.1136/annrheumdis-2014-205577 25115449

[B18] Clay P. G. (2002). The abacavir hypersensitivity reaction: a review. Clin. Ther. 24 (10), 1502–1514. 10.1016/s0149-2918(02)80057-1 12462283

[B19] Correia O. Delgado L. Barbosa I. L. Campilho F. Fleming-Torrinha J. (2002). Increased interleukin 10, tumor necrosis factor alpha, and interleukin 6 levels in blister fluid of toxic epidermal necrolysis. J. Am. Acad. Dermatol. 47 (1), 58–62. 10.1067/mjd.2002.120473 12077582

[B20] Daly A. K. Donaldson P. T. Bhatnagar P. Shen Y. Pe’er I. Floratos A. (2009). HLA-B*5701 genotype is a major determinant of drug-induced liver injury due to flucloxacillin. Nat. Genet. 41 (7), 816–819. 10.1038/ng.379 19483685

[B21] Dash C. H. (1975). Penicillin allergy and the cephalosporins. J. Antimicrob. Chemother. 1 (Suppl. 3), 107–118. 10.1093/jac/1.suppl_3.107 1201975

[B22] Dias de Castro E. Leblanc A. Sarmento A. Cernadas J. R. (2015). An unusual case of delayed-type hypersensitivity to ceftriaxone and meropenem. Eur. Ann. Allergy Clin. Immunol. 47 (6), 225–227. . 26549341

[B23] Duong T. A. Valeyrie-Allanore L. Wolkenstein P. Chosidow O. (2017). Severe cutaneous adverse reactions to drugs. Lancet. 390 (10106), 1996–2011. 10.1016/S0140-6736(16)30378-6 28476287

[B24] El-Ghaiesh S. Monshi M. M. Whitaker P. Jenkins R. Meng X. Farrell J. (2012). Characterization of the antigen specificity of T-cell clones from piperacillin-hypersensitive patients with cystic fibrosis. J. Pharmacol. Exp. Ther. 341 (3), 597–610. 10.1124/jpet.111.190900 22371438 PMC3362878

[B25] Empedrad R. Darter A. L. Earl H. S. Gruchalla R. S. (2003a). Nonirritating intradermal skin test concentrations for commonly prescribed antibiotics. J. Allergy Clin. Immunol. 112 (3), 629–630. 10.1016/s0091-6749(03)01783-4 13679828

[B26] Empedrad R. Darter A. L. Earl H. S. Gruchalla R. S. (2003b). Nonirritating intradermal skin test concentrations for commonly prescribed antibiotics. J. Allergy Clin. Immunol. 112 (3), 629–630. 10.1016/s0091-6749(03)01783-4 13679828

[B27] Gaeta F. Valluzzi R. L. Alonzi C. Maggioletti M. Caruso C. Romano A. (2015). Tolerability of aztreonam and carbapenems in patients with IgE-mediated hypersensitivity to penicillins. J. Allergy Clin. Immunol. 135 (4), 972–976. 10.1016/j.jaci.2014.10.011 25457154

[B28] Hama N. Nishimura K. Hasegawa A. Yuki A. Kume H. Adachi J. (2019). Galectin-7 as a potential biomarker of Stevens-Johnson syndrome/toxic epidermal necrolysis: identification by targeted proteomics using causative drug-exposed peripheral blood cells. J. Allergy Clin. Immunol. Pract. 7 (8), 2894–2897.e7. 10.1016/j.jaip.2019.05.002 31100551

[B29] Hari Y. Frutig-Schnyder K. Hurni M. Yawalkar N. Zanni M. P. Schnyder B. (2001). T cell involvement in cutaneous drug eruptions. Clin. Exp. Allergy. 31 (9), 1398–1408. 10.1046/j.1365-2222.2001.01164.x 11591190

[B30] Heinzerling L. Mari A. Bergmann K.-C. Bresciani M. Burbach G. Darsow U. (2013). The skin prick test–European standards. Clin. Transl. Allergy. 3 (1), 3 10.1186/2045-7022-3-3 23369181 PMC3565910

[B31] Hjortlund J. Mortz C. Skov P. Eller E. Poulsen J. M. Borch J. E. (2012). One-week oral challenge with penicillin in diagnosis of penicillin allergy. Acta Derm. Venereol. 92 (3), 307–312. 10.2340/00015555-1254 22170236

[B32] Hung S. I. Chung W. H. Liou L.-B. Chu C. C. Lin M. Huang H. P. (2005). HLA-B*5801 allele as a genetic marker for severe cutaneous adverse reactions caused by allopurinol. Proc. Natl. Acad. Sci. U.S.A. 102 (11), 4134–4139. 10.1073/pnas.0409500102 15743917 PMC554812

[B33] Illing P. T. Vivian J. P. Dudek N. L. Kostenko L. Chen Z. Bharadwaj M. (2012). Immune self-reactivity triggered by drug-modified HLA-peptide repertoire. Nature. 486 (7404), 554–558. 10.1038/nature11147 22722860

[B34] Johansen J. D. Aalto-Korte K. Agner T. Andersen K. E. Bircher A. Bruze M. (2015). European Society of Contact Dermatitis guideline for diagnostic patch testing-recommendations on best practice. Contact Dermatitis. 73 (4), 195–221. 10.1111/cod.12432 26179009

[B35] Kardaun S. H. Sidoroff A. Valeyrie-Allanore L. Halevy S. Davidovici B. B. Mockenhaupt M. (2007). Variability in the clinical pattern of cutaneous side-effects of drugs with systemic symptoms: does a DRESS syndrome really exist? Br. J. Dermatol. 156 (3), 609–611. 10.1111/j.1365-2133.2006.07704.x 17300272

[B36] Kauppinen K. (1991). Fixed drug eruptions and oral rechallenge. Cleve. Clin. J. Med. 58 (1), 64–65. 10.3949/ccjm.58.1.64 1829986

[B37] Keane N. M. Roberts S. G. Almeida C. A. Krishnan T. Chopra A. Demaine E. (2012). High-avidity, high-IFNgamma-producing CD8 T-cell responses following immune selection during HIV-1 infection. Immunol. Cell Biol. 90 (2), 224–234. 10.1038/icb.2011.34 21577229 PMC3173576

[B38] Khalil G. El-Sabban M. Al-Ghadban S. Azzi S. Shamra S. Khalifé S. (2008). Cytokine expression profile of sensitized human T lymphocytes following *in vitro* stimulation with amoxicillin. Eur. Cytokine Netw. 19 (3), 131–141. 10.1684/ecn.2008.0132 18775806

[B39] Kim J. Y. Sohn K. H. Song W. J. Kang H. R. (2013). A case of drug reaction with eosinophilia and systemic symptoms induced by ethambutol with early features resembling Stevens-Johnson syndrome. Acta Derm. Venereol. 93 (6), 753–754. 10.2340/00015555-1600 23584150

[B40] Komatsu-Fujii T. Kaneko S. Chinuki Y. Suyama Y. Ohta M. Niihara H. (2017). Serum TARC levels are strongly correlated with blood eosinophil count in patients with drug eruptions. Allergol. Int. 66 (1), 116–122. 10.1016/j.alit.2016.06.003 27497618

[B41] Komatsu-Fujii T. Chinuki Y. Niihara H. Hayashida K. Ohta M. Okazaki R. (2018). The thymus and activation-regulated chemokine (TARC) level in serum at an early stage of a drug eruption is a prognostic biomarker of severity of systemic inflammation. Allergol. Int. 67 (1), 90–95. 10.1016/j.alit.2017.06.001 28648978

[B42] Konvinse K. C. Phillips E. J. White K. D. Trubiano J. A. (2016). Old dog begging for new tricks: current practices and future directions in the diagnosis of delayed antimicrobial hypersensitivity. Curr. Opin. Infect. Dis. 29 (6), 561–576. 10.1097/QCO.0000000000000323 27753687 PMC5113146

[B43] Konvinse K. C. Trubiano J. A. Pavlos R. James I. Shaffer C. M. Bejan C. A. (2019). HLA-A*32:01 is strongly associated with vancomycin-induced drug reaction with eosinophilia and systemic symptoms. J. Allergy Clin. Immunol. 144 (1), 183–192. 10.1016/j.jaci.2019.01.045 30776417 PMC6612297

[B44] Lammintausta K. Kortekangas-Savolainen O. (2005). The usefulness of skin tests to prove drug hypersensitivity. Br. J. Dermatol. 152 (5), 968–974. 10.1111/j.1365-2133.2005.06429.x 15888154

[B45] Lochmatter P. Beeler A. Kawabata T. T. Gerber B. O. Pichler W. J. (2009). Drug-specific *in vitro* release of IL-2, IL-5, IL-13 and IFN-gamma in patients with delayed-type drug hypersensitivity. Allergy. 64 (9), 1269–1278. 10.1111/j.1398-9995.2009.01985.x 19254289

[B46] Lonjou C. Thomas L. Borot N. Ledger N. de Toma C. LeLouet H. (2006). A marker for Stevens-Johnson syndromeethnicity matters. Pharmacogenomics J. 6 (4), 265–268. 10.1038/sj.tpj.6500356 16415921

[B47] Makris M. P. Koulouris S. Kalogeromitros D. (2010). Nonimmediate systemic hypersensitivity reaction to beta-lactam intradermal tests. J Investig. Allergol. Clin. Immunol. 20 (7), 630–631. . 21314013

[B48] Mallal S. Nolan D. Witt C. Masel G. Martin A. M. Moore C. (2002). Association between presence of HLA-B*5701, HLA-DR7, and HLA-DQ3 and hypersensitivity to HIV-1 reverse-transcriptase inhibitor abacavir. Lancet. 359 (9308), 727–732. 10.1016/s0140-6736(02)07873-x 11888582

[B49] Mallal S. Phillips E. Carosi G. Molina J. M. Workman C. Tomazic J. (2008). HLA-B*5701 screening for hypersensitivity to abacavir. N. Engl. J. Med. 358 (6), 568–579. 10.1056/NEJMoa0706135 18256392

[B50] Mill C. Primeau M. N. Medoff E. Lejtenyi C. O’Keefe A. Netchiporouk E. (2016). Assessing the diagnostic properties of a graded oral provocation challenge for the diagnosis of immediate and nonimmediate reactions to amoxicillin in children. JAMA Pediatr. 170 (6), e160033 10.1001/jamapediatrics.2016.0033 27043788

[B51] Murata J. Abe R. Shimizu H. (2008). Increased soluble Fas ligand levels in patients with Stevens-Johnson syndrome and toxic epidermal necrolysis preceding skin detachment. J. Allergy Clin. Immunol. 122 (5), 992–1000. 10.1016/j.jaci.2008.06.013 18692887

[B52] Nagao-Dias A. T. Teixeira F. M. Coelho H. L. (2009). Diagnosing immune-mediated reactions to drugs. Allergol. Immunopathol. 37 (2), 98–104. 10.1016/s0301-0546(09)71112-7 19445867

[B53] Nyfeler B. Pichler W. J. (1997). The lymphocyte transformation test for the diagnosis of drug allergy: sensitivity and specificity. Clin. Exp. Allergy. 27 (2), 175–181. 9061217

[B54] Ogawa K. Morito H. Hasegawa A. Daikoku N. Miyagawa F. Okazaki A. (2013). Identification of thymus and activation-regulated chemokine (TARC/CCL17) as a potential marker for early indication of disease and prediction of disease activity in drug-induced hypersensitivity syndrome (DIHS)/drug rash with eosinophilia and systemic symptoms (DRESS). J. Dermatol. Sci. 69 (1), 38–43. 10.1016/j.jdermsci.2012.10.002 23141052

[B55] Osawa J. Naito S. Aihara M. Kitamura K. Ikezawa Z. Nakajima H. (1990). Evaluation of skin test reactions in patients with non-immediate type drug eruptions. J. Dermatol. 17 (4), 235–239. 10.1111/j.1346-8138.1990.tb01631.x 2142173

[B56] Ostrov D. A. Grant B. J. Pompeu Y. A. Sidney J. Harndahl M. Southwood S. (2012). Drug hypersensitivity caused by alteration of the MHC-presented self-peptide repertoire. Proc. Natl. Acad. Sci. U.S.A. 109 (25), 9959–9964. 10.1073/pnas.1207934109 22645359 PMC3382472

[B57] Ozkaya-Bayazit E. Bayazit H. Ozarmagan G. (1999). Topical provocation in 27 cases of cotrimoxazole-induced fixed drug eruption. Contact Dermatitis. 41 (4), 185–189. 10.1111/j.1600-0536.1999.tb06127.x 10515095

[B58] Padovan E. Mauri-Hellweg D. Pichler W. J. Weltzien H. U. (1996). T cell recognition of penicillin G: structural features determining antigenic specificity. Eur. J. Immunol. 26 (1), 42–48. 10.1002/eji.1830260107 8566082

[B59] Peter J. G. Lehloenya R. Dlamini S. Risma K. White K. D. Konvinse K. C. (2017). Severe delayed cutaneous and systemic reactions to drugs: a global perspective on the science and art of current practice. J. Allergy Clin. Immunol. Pract. 5 (3), 547–563. 10.1016/j.jaip.2017.01.025 28483310 PMC5424615

[B60] Phillips E. J. Sullivan J. R. Knowles S. R. Shear N. H. (2002). Utility of patch testing in patients with hypersensitivity syndromes associated with abacavir. AIDS. 16 (16), 2223–2225. 10.1097/00002030-200211080-00017 12409746

[B61] Phillips E. J. Bigliardi P. Bircher A. J. Broyles A. Chang Y. S. Chung W. H. (2019). Controversies in drug allergy: testing for delayed reactions. J. Allergy Clin. Immunol. 143 (1), 66–73. 10.1016/j.jaci.2018.10.030 30573342 PMC6429556

[B62] Phillips E. J. (2016). Classifying ADRs–does dose matter? Br. J. Clin. Pharmacol. 81 (1), 10–12. 10.1111/bcp.12749 26286675 PMC4693569

[B63] Picard M. Robitaille G. Karam F. Daigle J. M. Bédard F. Biron É. (2019). Cross-reactivity to cephalosporins and carbapenems in penicillin-allergic patients: two systematic reviews and meta-analyses. J. Allergy Clin. Immunol. Pract. 7 (8), 2722–2738. 10.1016/j.jaip.2019.05.038 31170539

[B64] Pichler W. J. Hausmann O. (2016). Classification of drug hypersensitivity into allergic, p-i, and pseudo-allergic forms. Int. Arch. Allergy Immunol. 171 (3–4), 166–179. 10.1159/000453265 27960170

[B65] Pichler W. J. Tilch J. (2004). The lymphocyte transformation test in the diagnosis of drug hypersensitivity. Allergy. 59 (8), 809–820. 10.1111/j.1398-9995.2004.00547.x 15230812

[B66] Pichler W. J. Watkins S. (2014). Interaction of small molecules with specific immune receptors: the p-i concept and its consequences. Curr. Immunol. Rev. 10 (1), 7–18. 10.2174/1573395510666140407212357

[B67] Polak M. E. Belgi G. McGuire C. Pickard C. Healy E. Friedmann P. S. (2013). *In vitro* diagnostic assays are effective during the acute phase of delayed-type drug hypersensitivity reactions. Br. J. Dermatol. 168 (3), 539–549. 10.1111/bjd.12109 23106791

[B68] Porebski G. Pecaric-Petkovic T. Groux-Keller M. Bosak M. Kawabata T. T. Pichler W. J. (2013). *In vitro* drug causality assessment in Stevens-Johnson syndrome–alternatives for lymphocyte transformation test. Clin. Exp. Allergy. 43 (9), 1027–1037. 10.1111/cea.12145 23957338

[B69] Porebski G. Czarnobilska E. Bosak M. (2015). Cytotoxicbased assays in delayed drug hypersensitivity reactions induced by antiepileptic drugs. Pol. Arch. Med. Wewn. 125 (11), 823–834. 10.20452/pamw.3160 26445768

[B70] Posadas S. J. Padial A. Torres M. J. Mayorga C. Leyva L. Sanchez E. (2002). Delayed reactions to drugs show levels of perforin, granzyme B, and Fas-L to be related to disease severity. J. Allergy Clin. Immunol. 109 (1), 155–161. 10.1067/mai.2002.120563 11799383

[B71] Rive C. M. Bourke J. Phillips E. J. (2013). Testing for drug hypersensitivity syndromes. Clin. Biochem. Rev. 34 (1), 15–38. . 23592889 PMC3626363

[B72] Romano A. Gaeta F. Valluzzi R. L. Maggioletti M. Caruso C. Quaratino D. (2016). Cross-reactivity and tolerability of aztreonam and cephalosporins in subjects with a T cell-mediated hypersensitivity to penicillins. J. Allergy Clin. Immunol. 138 (1), 179–186. 10.1016/j.jaci.2016.01.025 27016799

[B73] Rozieres A. Hennino A. Rodet K. Gutowski M. C. Gunera-Saad N. Berard F. (2009). Detection and quantification of drug-specific T cells in penicillin allergy. Allergy. 64 (4), 534–542. 10.1111/j.1398-9995.2008.01674.x 19154548

[B74] Saito N. Abe R. Yoshioka N. Murata J. Fujita Y. Shimizu H. (2012). Prolonged elevation of serum granulysin in drug-induced hypersensitivity syndrome. Br. J. Dermatol. 167 (2), 452–453. 10.1111/j.1365-2133.2012.10921.x 22384988

[B75] Sala Cunill A. Labrador-Horrillo M. Guilarte M. Luengo O. Cardona V. (2011). Generalised delayed desquamative exanthema after intradermal testing with betalactam antibiotics. Allergy. 66 (5), 702–703. 10.1111/j.1398-9995.2010.02495.x 21470241

[B76] Shear N. H. Dodiuk-Gad R. P. (2019). Advances in diagnosis and management of cutaneous adverse drug reactions., Vol. 1 Singapore: ADIS, 307.

[B77] Shiina T. Inoko H. Kulski J. K. (2004). An update of the HLA genomic region, locus information and disease associations: 2004. Tissue Antigens. 64 (6), 631–649. 10.1111/j.1399-0039.2004.00327.x 15546336

[B78] Shiohara T. Mizukawa Y. Aoyama Y. (2015). Monitoring the acute response in severe hypersensitivity reactions to drugs. Curr. Opin. Allergy Clin. Immunol. 15 (4), 294–299. 10.1097/ACI.0000000000000180 26110678

[B79] Sidoroff A. Halevy S. Bavinck J. N. B. Vaillant L. Roujeau J. C. (2001). Acute generalized exanthematous pustulosis (AGEP)–a clinical reaction pattern. J. Cutan. Pathol. 28 (3), 113–119. 10.1034/j.1600-0560.2001.028003113.x 11168761

[B80] Su S. C. Hung S. I. Fan W. L. Dao R. L. Chung W. H. (2016). Severe cutaneous adverse reactions: the pharmacogenomics from research to clinical implementation. Int. J. Mol. Sci. 17 (11), 1890 10.3390/ijms17111890 PMC5133889 27854302

[B81] Su S. C. Mockenhaupt M. Wolkenstein P. Dunant A. Le Gouvello S. Chen C. B. (2017). Interleukin-15 is associated with severity and mortality in Stevens-Johnson syndrome/toxic epidermal necrolysis. J. Invest. Dermatol. 137 (5), 1065–1073. 10.1016/j.jid.2016.11.034 28011147

[B82] Suthumchai N. Srinoulprasert Y. Thantiworasit P. Rerknimitr P. Tuchinda P. Chularojanamontri L. (2018). The measurement of drug-induced interferon gamma-releasing cells and lymphocyte proliferation in severe cutaneous adverse reactions. J. Eur. Acad. Dermatol. Venereol. 32 (6), 992–998. 10.1111/jdv.14890 29478292

[B83] Syrigou E. Zande M. Grapsa D. Syrigos K. (2016). Severe delayed skin reaction during intradermal testing with beta-lactam antibiotics. J. Allergy Clin. Immunol. Pract. 4 (1), 158–159. 10.1016/j.jaip.2015.07.018 26320832

[B84] Teraki Y. Kawabe M. Izaki S. (2013). Possible role of TH17 cells in the pathogenesis of Stevens-Johnson syndrome and toxic epidermal necrolysis. J. Allergy Clin. Immunol. 131 (3), 907–909. 10.1016/j.jaci.2012.08.042 23083672

[B85] Tomida E. Kato Y. Ozawa H. Hasegawa H. Ishii N. Hashimoto T. (2016). Causative drug detection by drug-induced lymphocyte stimulation test in drug-induced linear IgA bullous dermatosis. Br. J. Dermatol. 175, 1106–1108. 10.1111/bjd.14069 26265104

[B86] Torres M. J. Sánchez-Sabaté E. Alvarez J. Mayorga C. Fernández J. Padial A. (2004). Skin test evaluation in nonimmediate allergic reactions to penicillins. Allergy. 59 (2), 219–224. 10.1046/j.1398-9995.2003.00308.x 14763937

[B87] Trubiano J. A. Adkinson N. F. Phillips E. J. (2017a). Penicillin allergy is not necessarily forever. J. Am. Med. Assoc. 318 (1), 82–83. 10.1001/jama.2017.6510 PMC5935455 28672303

[B88] Trubiano J. A. Redwood A. Strautins K. Pavlos R. Woolnough E. Chang C. C. (2017b). Drug-specific upregulation of CD137 on CD8+ T cells aids in the diagnosis of multiple antibiotic toxic epidermal necrolysis. J. Allergy Clin. Immunol. Pract. 5 (3), 823–826. 10.1016/j.jaip.2016.09.043 27888029 PMC5436601

[B89] Trubiano J. A. Strautins K. Redwood A. J. Pavlos R. Konvinse K. C. Aung A. K. (2018). The combined utility of ex vivo IFN-gamma release enzyme-linked ImmunoSpot assay and in vivo skin testing in patients with antibiotic-associated severe cutaneous adverse reactions. J. Allergy Clin. Immunol. Pract. 6 (4), 1287–1296 e1. 10.1016/j.jaip.2017.09.004 29100867 PMC5930120

[B90] Trubiano J. A. Chua K. Y. L. Holmes N. E. Douglas A. P. Mouhtouris E. Goh M. (2020). Safety of cephalosporins in penicillin class severe delayed hypersensitivity reactions. J Allergy Clin Immunol Pract. 8 (3), 1142–1146.e4. 10.1016/j.jaip.2019.10.005 31678298 PMC7064395

[B91] Trubiano J. Phillips E. (2013). Antimicrobial stewardship’s new weapon? A review of antibiotic allergy and pathways to ‘de-labeling’. Curr. Opin. Infect. Dis. 26 (6), 526–537. 10.1097/QCO.0000000000000006 24126717 PMC3862073

[B92] Watts T. J. (2017). Severe delayed-type hypersensitivity to chloramphenicol with systemic reactivation during intradermal testing. Ann. Allergy Asthma Immunol. 118 (5), 644–645. 10.1016/j.anai.2017.03.004 28477797

[B93] Weinborn M. Barbaud A. Truchetet F. Beurey P. Germain L. Cribier B. (2016). Histopathological study of six types of adverse cutaneous drug reactions using granulysin expression. Int. J. Dermatol. 55 (11), 1225–1233. 10.1111/ijd.13350 27421110

[B94] White K. D. Chung W. H. Hung S. I. Mallal S. Phillips E. J. (2015). Evolving models of the immunopathogenesis of T cell-mediated drug allergy: the role of host, pathogens, and drug response. J. Allergy Clin. Immunol. 136 (2), 219–235; quiz 235 10.1016/j.jaci.2015.05.050 26254049 PMC4577472

[B95] Wolf R. Tuzun Y. (2015). Baboon syndrome and toxic erythema of chemotherapy: fold (intertriginous) dermatoses. Clin. Dermatol. 33 (4), 462–465. 10.1016/j.clindermatol.2015.04.008 26051062

[B96] Wolkenstein P. Chosidow O. Fléchet M. L. Robbiola O. Paul M. Dumé L. (1996). Patch testing in severe cutaneous adverse drug reactions, including Stevens-Johnson syndrome and toxic epidermal necrolysis. Contact Dermatitis. 35 (4), 234–236. 10.1111/j.1600-0536.1996.tb02364.x 8957644

[B97] Yoon S. Y. Baek S. H. Kim S. Lee Y. S. Lee T. Bae Y. J. (2013). Serum procalcitonin as a biomarker differentiating delayed-type drug hypersensitivity from systemic bacterial infection. J. Allergy Clin. Immunol. 132 (4), 981–983. 10.1016/j.jaci.2013.04.038 23768571

[B98] Zawodniak A. Lochmatter P. Yerly D. Kawabata T. Lerch M. Yawalkar N. (2010). *In vitro* detection of cytotoxic T and NK cells in peripheral blood of patients with various drug-induced skin diseases. Allergy. 65 (3), 376–384. 10.1111/j.1398-9995.2009.02180.x 19793058

[B99] Zhang F. R. Liu H. Irwanto A. Fu X. A. Li Y. Yu G. Q. (2013) HLA-B*13:01 and the dapsone hypersensitivity syndrome. N. Engl. J. Med. 369 (17), 1620–1628. 10.1056/NEJMoa1213096 24152261

